# Genomic and evolutionary factors influencing the prediction accuracy of optimal growth temperature in prokaryotes

**DOI:** 10.1128/msystems.00062-26

**Published:** 2026-04-03

**Authors:** Seiji Toki, Motomu Matsui, Kento Tominaga, Takao K. Suzuki, Takashi Tsuchimatsu, Wataru Iwasaki

**Affiliations:** 1 Department of Biological Sciences, The University of Tokyo, Graduate School of Science 13143, Bunkyo-ku, Tokyo, Japan; 2 Department of Integrated Biosciences, Graduate School of Frontier Sciences, The University of Tokyo 34811 , Kashiwa, Chiba, Japan; 3 Institute for Chemical Research, Kyoto University 12918, Kyoto, Japan; 4 Department of Natural Environmental Studies, Graduate School of Frontier Sciences, The University of Tokyo, Kashiwa, Chiba, Japan; 5 Department of Biochemistry and Systems Biomedicine, Juntendo University Graduate School of Medicine 231191 , Bunkyo-ku, Tokyo, Japan; 6 Atmosphere and Ocean Research Institute, The University of Tokyo 74024 , Kashiwa, Chiba, Japan; CNRS Delegation Bretagne et Pays de Loire, Nantes, France

**Keywords:** optimal growth temperature, psychrophiles, evolution, machine learning

## Abstract

Bacteria and archaea have evolved diverse genomic adaptations to thrive across various temperatures. These adaptations include genome sequence optimizations, such as increased GC content in rRNA and tRNA, shifts in codon and amino acid usage, and the acquisition of functional genes conferring adaptation for specific temperatures. Since the experimental determination of optimal growth temperatures (OGTs) is only possible for cultured species, predicting OGT from genomic information has become increasingly important given the exponential increase in genomic data. Although previous studies developed prediction models integrating multiple features based on genome composition using machine learning, the accuracy was variable depending on the target species, with models performing well for thermophiles but less accurately for psychrophiles. In this study, we curated the OGTs and genomic data of 2,869 bacterial species to develop a novel prediction model incorporating features reflecting genomic adaptation toward lower temperatures. We found that species with rapid OGT shifts from their ancestors, including psychrophiles, showed less accuracy in genome composition-based models. Incorporating the gene presence/absence information associated with the rapid changes in OGT improved the prediction accuracy for psychrophiles. We also observed that OGT in archaea is phylogenetically more conserved than in bacteria, which may lead to the long-term optimization of the genome composition and explain high predictability of OGT in archaea. These findings highlight the importance of integrating long- and short-term evolutionary adaptations for phenotype prediction models.

**IMPORTANCE:**

Prediction of optimal growth temperature (OGT) from genomic data allows for characterizing uncultivated microbes. This study developed a novel OGT prediction model by curating OGT and genomic data of 2,869 bacterial species, demonstrating that the prediction accuracy was improved by incorporating not only genome composition features reflecting long-term temperature adaptation but also the presence/absence information of genes conferring short-term adaptation, particularly for species undergoing rapid OGT shifts. Our study provides a framework for improving phenotype prediction models by integrating long- and short-term evolutionary factors, which may also apply to other microbial physiological phenotypes.

## INTRODUCTION

Bacteria and archaea have adapted to a wide range of temperatures from below 0°C to over 100°C (1, 2), and their genomes have undergone diverse evolutionary modifications in response to their habitat temperatures. In general, bacterial thermal adaptation strategies can be classified into two major types: optimization of genome sequences (3) and the gain of genes that confer specific functions (4). Since genome sequences fundamentally determine the stability of nucleic acid secondary structures and the folding of the encoding proteomes, they should be optimized to specific habitat temperatures. For example, the GC content of rRNA and tRNA is elevated, and the ratio of charged to polar amino acids increases in thermophiles (5–7). In addition, synonymous codon usage for arginine varies in response to the species’ habitat (8). The gain of genes also facilitates bacterial adaptation to specific temperatures. Some thermophiles possess reverse gyrases that introduce positive supercoil and stabilize DNA under high temperatures (9). Similarly, psychrophiles often possess chaperones that facilitate proper RNA folding in cold environments and antifreeze proteins that inhibit the formation of ice crystals inside the cells (1).

Although growth temperature is one of the most fundamental parameters of prokaryotic physiology, the experimental determination of prokaryotes’ optimal growth temperature (OGT) is laborious and applicable only to cultured species (10). Therefore, predicting OGT from genomic information has become increasingly important given the exponential increase in genomic data from cultivated and uncultivated prokaryotes in recent years (11, 12). For the prediction of OGT, the information on genome composition (i.e., base composition, codon usages, and amino acids composition) has been widely adopted because it does not require functional gene annotation and is applicable across a broad range of taxa. As a simple and reliable indicator of OGT, a previous study demonstrated that the fraction of seven amino acids—isoleucine, valine, tyrosine, tryptophan, arginine, glutamic acid, and leucine (abbreviated as IVYWREL)—shows a strong correlation with OGT (13). Recently, machine learning has been incorporated to improve the prediction, integrating many genome-derived features, such as tRNA and rRNA base composition, codon usage, and amino acid composition, leading to the development of highly accurate prediction models (12, 14). These studies demonstrate that OGT can be generally predictable based on genome composition.

Despite such methodological advancement in predicting OGT, its accuracy based on genome composition was variable depending on the target. While the model showed an improved fit for thermophiles, an accurate prediction of OGT for psychrophiles based on genomic data poses challenges, possibly because of the underrepresentation of species and/or features associated with the adaptation to low temperatures (12, 15). Furthermore, a previous study reported higher correlations between genomic features and OGT in archaea than in bacteria, resulting in higher predictability of OGT in archaea (12). However, the reasons for the differences in prediction accuracy between targets are largely unexplored.

In this study, we aimed to clarify the reasons for the difficulty in the OGT prediction from prokaryotic genomes, especially in psychrophiles, and provide a framework for improving accuracy. Using the curated OGT and genomic data of 2,869 bacterial species, we developed a novel prediction model incorporating features reflecting the genomic adaptation toward lower temperatures. We found that the model based on genome composition poses a challenge in predicting the OGT of species whose OGT has rapidly changed from their ancestors, including psychrophiles, and that the prediction accuracy of OGT in psychrophiles was improved by incorporating the gene presence/absence information associated with the rapid changes in OGT. Finally, we discovered that OGT in archaea is phylogenetically more conserved than in bacteria, which may lead to the long-term optimization of the genome composition and explain why the predictability of OGT is high in archaea. These results highlight the importance of considering traits’ long- and short-term evolution for developing highly accurate phenotype prediction models.

## RESULTS

### Constructing an OGT prediction model based on genome composition

**Fig 1:**
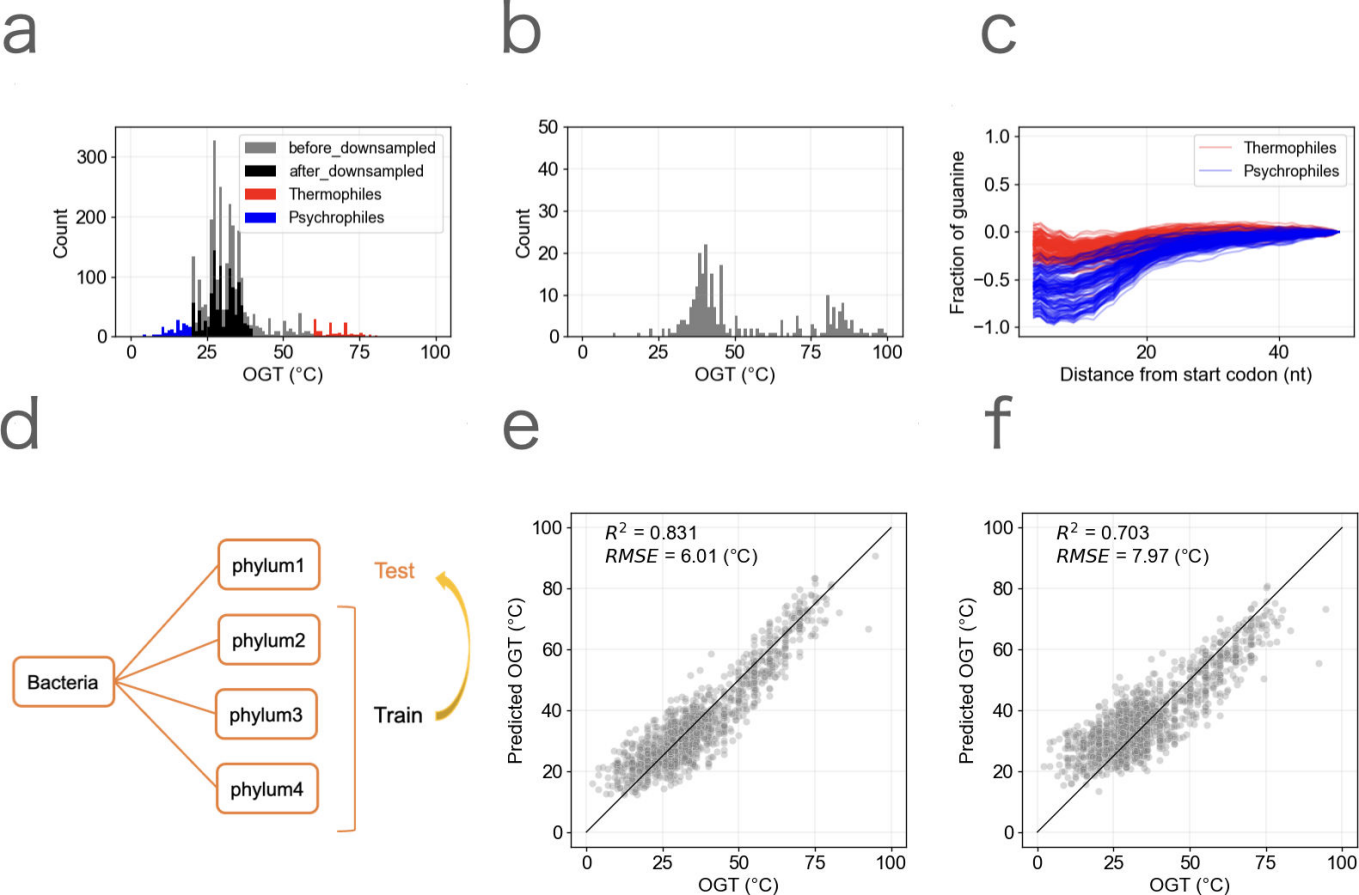
Constructing an OGT prediction model based on genome composition. (**a**) The distribution of OGT of bacteria (2,869 species). Red and blue indicate thermophiles (OGT ≥ 60°C) and psychrophiles (OGT < 20°C), respectively. Black indicates the distribution after downsampling mesophiles (20°C ≤ OGT < 40°C). (**b**) The distribution of OGT of archaea (262 species). (**c**) The fraction of guanine in the downstream region of the start codon. The fraction was normalized at 50 nt downstream of the start codon by taking the logarithm to base 2. The red and blue lines indicate thermophiles and psychrophiles, respectively. (**d**) Overview of the leave-one-phylum-out cross-validation. (**e and f**) The comparison of the prediction models based on genome composition. Each dot represents one bacterial species. The predictions based on genome composition using Lasso regression (**e**) and the prediction using the model of Li et al. (14) (replicated in this study) (**f**) are shown.

We first curated the OGT data and genomes of corresponding species from public databases. After merging OGT from three databases (16–18) and the corresponding genomes of GTDB (19–22), OGT and genome of 2,869 bacterial and 262 archaeal species were obtained (Fig. 1a and b). In this study, we defined thermophiles as species with OGT ≥ 60°C and psychrophiles as species with OGT < 20°C, according to the previous studies (23, 24). Because OGT was most frequent from 20 to 40°C (2,278 species; mesophiles) as previously reported (14), the model may overfit this temperature range and underestimate psychrophile features. We downsampled the data of in total 1,631 species (psychrophiles: 164 species, mesophiles: 1,040, thermophiles: 155 species) to verify this hypothesis by selecting one species per genus whose OGT is 20°C–40°C.

Next, we calculated genomic features used for OGT prediction. We first computed features similar to those used in previous studies (12, 14), including tRNA base composition, rRNA base composition, amino acid composition, and codon composition (Table S1). While the GC content of tRNA and rRNA is known to correlate with temperature (3, 5, 6), we also calculated the minimum free energy (MFE) of tRNA and rRNA that showed a stronger correlation with OGT than GC content (Fig. S1), suggesting that the MFE is the feature that better reflects the secondary structure of RNA. To improve the prediction of psychrophiles, we also calculated the features around the start codon because a stronger selection pressure would be exerted around the start codon of the mRNA sequence at lower temperatures, given the inhibition of the initiation of translation by the secondary structures (25, 26). Consistently, we found that the frequency of guanine significantly decreases in psychrophiles compared with thermophiles (Fig. 1c), incorporating the frequency of guanine and amino acids around the start codon for the prediction (21 features; Fig. S2).

We developed the OGT prediction model using the features described above. To verify if the model can predict the OGT of clades that are not used for the training data set, a leave-one-phylum-out cross-validation was performed, in which one phylum was used as the test data, and the rest were used as the training data (Fig. 1d). This method ensures that the model learns features that are commonly related to OGT across a wide range of lineages. We found that Lasso regression showed higher accuracy (*R^2^* = 0.831, RMSE = 6.01°C) than support vector regression (SVR) (*R^2^* = 0.768, RMSE = 7.03°C) (Fig. 1e; Fig. S3). Although one of the most accurate prediction models reported to date used SVR based on the amino acid 2-mer composition (14), the reproduced model was also less accurate compared to our models when evaluated using leave-one-phylum-out cross-validation (*R^2^* = 0.703, RMSE = 7.97°C; Fig. 1f). We also note that our model was shown the improved performance compared with the one by Sauer & Wang (12), likely due in part to the inclusion of additional features, such as amino acids 2-mer composition and features around the start codon (*R^2^* = 0.754 → 0.831).

Despite the improvement in overall accuracy, the accurate prediction of OGTs for psychrophiles remained challenging (mean absolute error = 7.1°C) compared with the accuracy of other temperature ranges (mean absolute error = 4.4°C). The prediction of OGTs for psychrophiles became worse without downsampling the data (mean absolute error = 8.0°C), without features around the start codon (mean absolute error = 7.7°C), or without both procedures (mean absolute error = 8.8°C). We also note that, if the downsampling was applied not only for mesophiles but also across the full temperature range, the prediction error of psychrophiles became worse (mean absolute error = 8.4°C). These results indicate that balancing the distribution of OGT in the training data set and incorporating features reflecting low-temperature adaptation were effective but not enough to resolve the issue fully.

**Fig 2:**
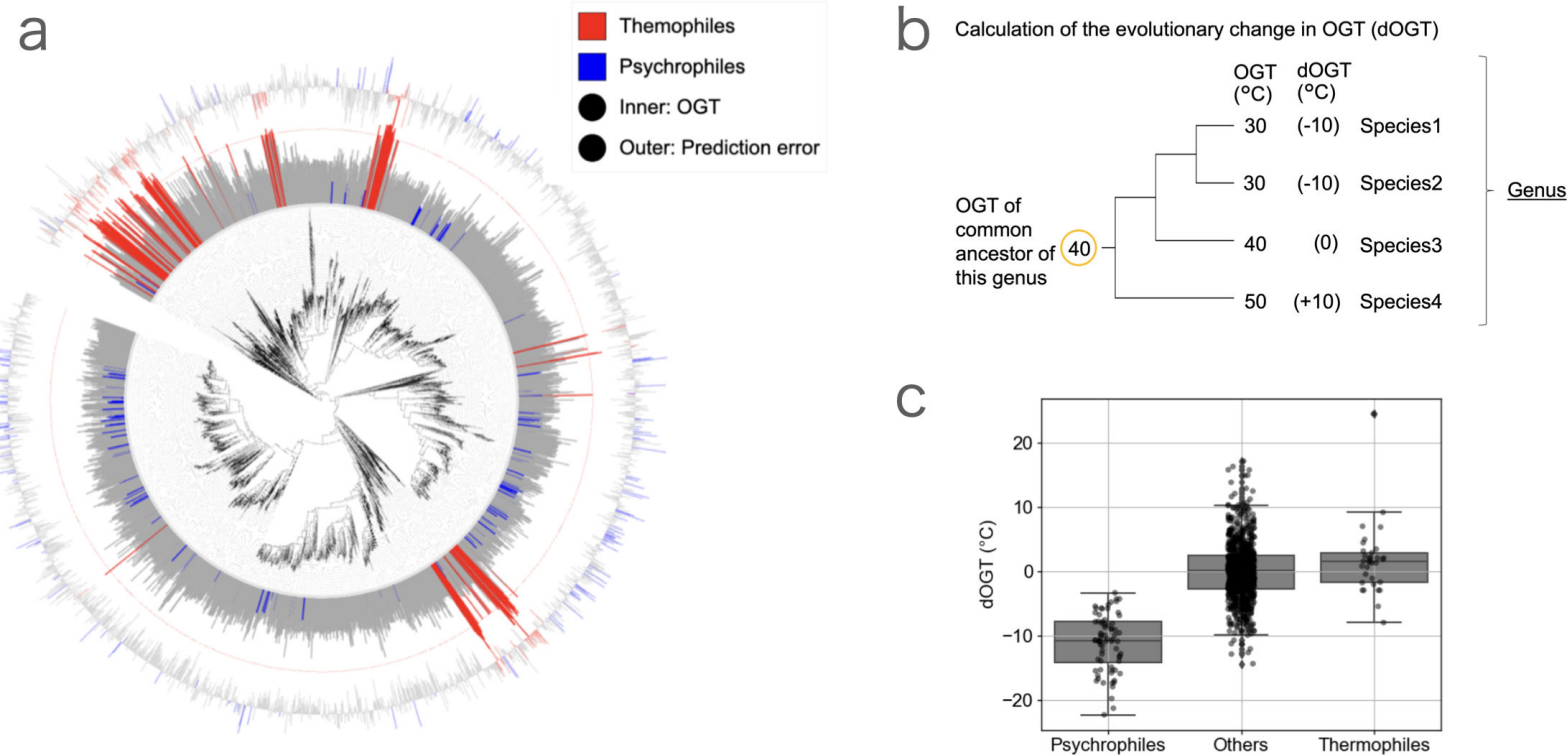
Calculation of the evolutionary change in OGT. (**a**) The distribution of OGT of bacteria with phylogenetic tree. The inner and outer bars indicate OGT and the prediction error of the model based on genome composition (Fig. 1d), respectively. Red and blue bars indicate thermophiles (OGT ≥ 60°C) and psychrophiles (OGT < 20°C), respectively. The phylogenetic tree was obtained from GTDB r207. (**b**) The scheme of calculating the evolutionary change in OGT (dOGT) of each species. OGT of the common ancestor of each genus was calculated, and dOGT was defined as OGT_current_ – OGT_ancestor_. (**c**) dOGT of psychrophiles (OGT < 20°C), thermophiles (OGT ≥ 60°C), and other bacteria. Horizontal lines indicate median, boxes include second and third quartiles, and whiskers extend to points that lie within 1.5 times of the interquartile range. The dOGT of psychrophiles was –11.0°C on average.

Next, we further investigated the factors causing inaccuracy in predicting the OGT of psychrophiles. We found that, while thermophiles were clustered in close clades (Blomberg’s *K* = 0.16), psychrophiles were relatively more sporadically distributed compared to thermophiles (Blomberg’s *K* = 0.04) in the phylogenetic tree (Fig. 2a) (27, 28). This result suggests that the OGT of psychrophiles may be more prone to change compared to those of thermophiles. We estimated the evolutionary change in OGT of each species from their most recent common ancestor of the genus to which the focal species belongs (dOGT; see detail in Materials and Methods) (Fig. 2b). All dOGT of psychrophiles were negative (mean dOGT = −11.0°C), supporting the recent decrease in the OGT of psychrophiles (Fig. 2c). In contrast, thermophilic species exhibit dOGT values close to zero, with an average dOGT of 1.7°C. It has been reported that the adaptation of amino acid composition requires the accumulation of numerous mutations and can take a long time (29, 30). Therefore, we reasoned that rapid alterations in the OGT of psychrophiles may underlie the inaccuracy in the genome composition-based prediction. To test this hypothesis, we evaluated how rapid alterations in the OGT affect the prediction result in the next section.

### The model based on genome composition cannot capture the diversity of OGT within the genus

**Fig 3:**
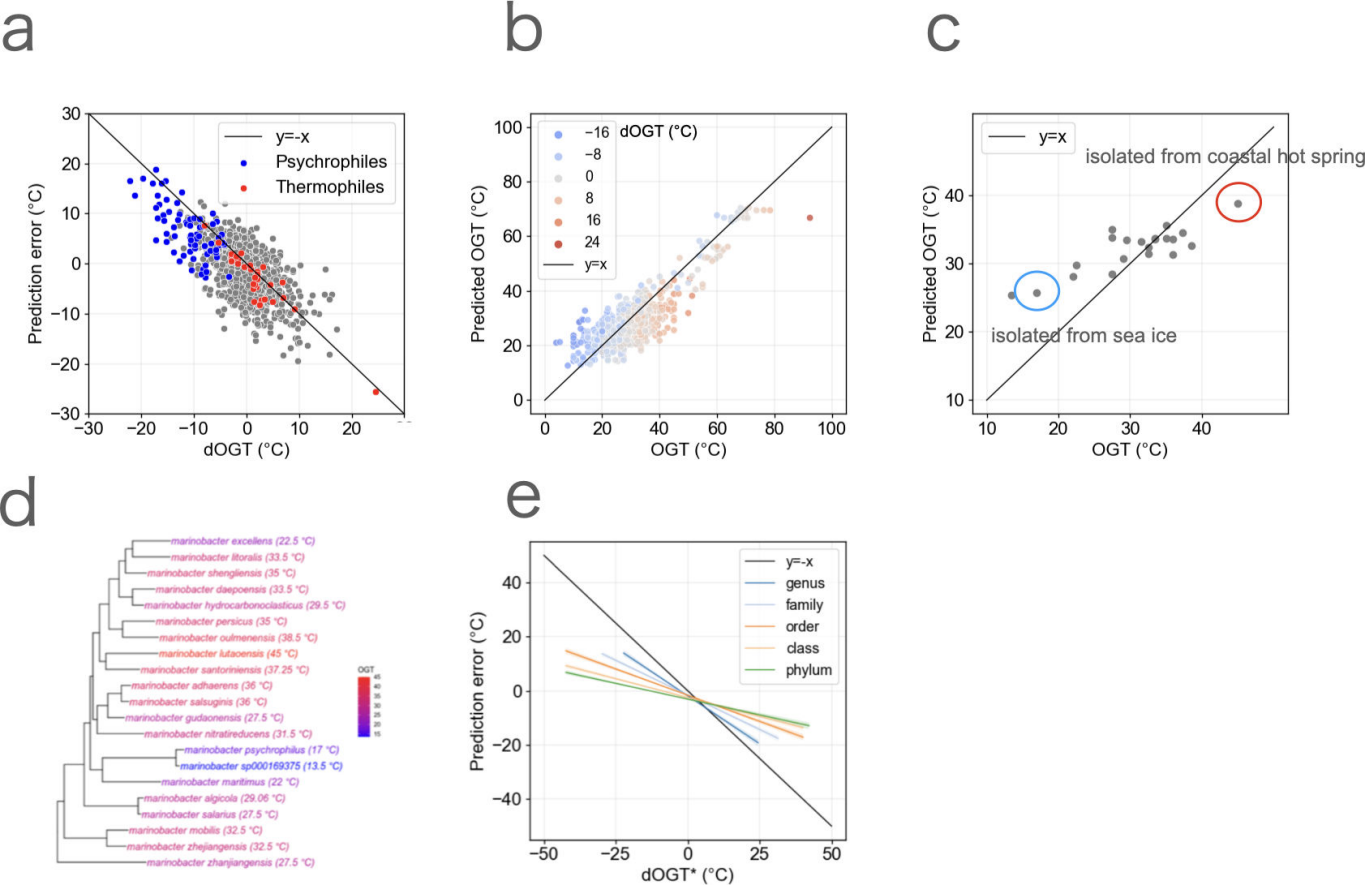
The model based on genome composition cannot fully capture the diversity of OGT within genera. (**a**) The relationship between dOGT and the prediction error (predicted OGT − observed OGT). Each point represents each species. The red and blue points represent thermophiles and psychrophiles, respectively. (**b**) The prediction result (Fig. 1d) is colored by dOGT. Blue and red indicate the species whose dOGT is under and over 0, respectively. (**c**) The prediction of OGT for *Marinobacter. Marinobacter psychrophilus* (red circle) and *Marinobacter lutaoensis* (blue circle) were isolated from sea ice and coastal hot spring, respectively. (**d**) The phylogenetic tree obtained from GTDB r207, with OGT of the genus *Marinobacter*. The color represents OGT. (**e**) Regression lines of the scatter plot showing the relationship between dOGT* (OGT changes from the common ancestor at the genus, family, order, class, and phylum levels) and the prediction error. The shade indicates the 95% confidence interval (CI) of the regression line.

We examined whether the model can predict the OGT of species that experienced rapid OGT alterations (Fig. 3a and b). We found that the predicted OGT of species with large dOGT tends to be distinct from the observed OGT and that the predicted OGT is close to the ancestral OGT, although this pattern may partly be due to the regression to the mean. For example, in *Marinobacter*, the observed OGT varies from 13°C (*Marinobacter psychrophilus*) to over 45°C (*Marinobacter lutaoensis*), while all predicted OGTs ranged from 25°C to 39°C (31, 32) (Fig. 3c and d).

If the genome composition adapts over time, the relationship between dOGT and prediction error should become weaker. To test this possibility, we investigated the evolutionary change in OGT in longer terms. Specifically, while dOGT was the change in OGT of each species from their most recent common ancestor of the genus, we estimated the evolutionary change in OGT from their most recent common ancestors at the family, order, class, and phylum levels (denoted as dOGT*). We found that the relationship between dOGT* and prediction error became weaker as the taxonomic scale of dOGT* became larger (genus, family, order, class, and phylum levels), suggesting that prediction error was strongly associated with more recent shift in OGT (Fig. 3e). These results demonstrate that the inaccuracy in predicting the OGT of psychrophiles based on genome composition is attributed to the relatively recent decrease in their OGT, leading to insufficient optimization of genome composition.

### Using the information on the presence and absence of genes to improve the model

The genome composition of psychrophiles is often not optimized, making it difficult to predict their OGT based solely on genomic information. Nonetheless, psychrophiles exhibit lower OGT than their close relatives, suggesting that additional mechanisms contribute to their thermal adaptation. To explore this possibility, we focus on the presence or absence of genes associated with habitat temperature. Gene acquisition and loss are major factors in prokaryotic thermal adaptation (1, 9), which has not been explicitly incorporated in previous studies (12, 14). Thus, we tested whether integrating gene presence/absence information could improve prediction accuracy for psychrophiles.

**Fig 4:**
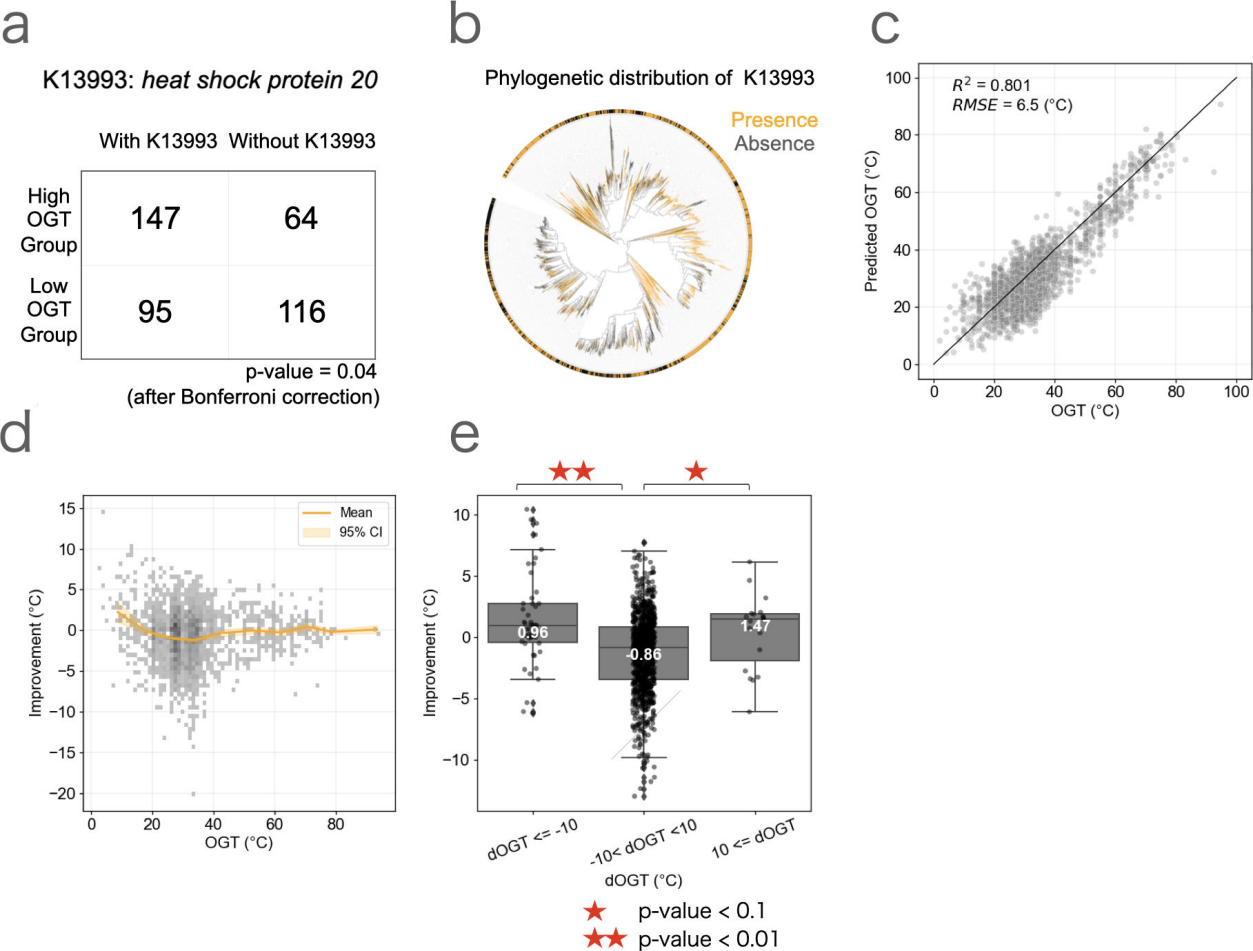
Inclusion of the gene presence/absence information improves the prediction accuracy. (**a**) A contingency table showing the association between the presence/absence of K13993 and OGT (*P* = 4.0 × 10^−6^ [0.04 after Bonferroni correction]: Fisher’s exact test). (**b**) The distribution of K13993 in the phylogenetic tree obtained from GTDB r207. Orange and black indicate presence and absence of K13993, respectively. (**c**) The prediction by incorporating the presence and absence of the 100 genes into the model based on genome composition (Fig. 1d). (**d**) The prediction improvement by incorporating the presence and absence of the 100 genes into the model based on genome composition (Fig. 1d). The orange line and shaded area represent the moving average and 95% confidence interval, respectively. (**e**) The relationship between dOGT and the improvement of prediction after incorporating the presence/absence information of the 100 OGT-associated genes into the model based on genome composition (Fig. 1d). Statistical significance was assessed using one-way ANOVA followed by Tukey’s post-hoc test. (* *P* < 0.1, ** *P* < 0.01). Horizontal lines indicate median, boxes include second and third quartiles, and whiskers extend to points that lie within 1.5 times of the interquartile range.

Genes specific to a few clades may not be suitable for general predictions of OGT, particularly for uncultured bacterial clades whose OGTs remain poorly studied. Therefore, we focused on OGT-associated genes conserved across various bacterial lineages and OGT ranges. For this purpose, we selected species with relatively high and low OGT within each genus. We categorized them into a “high OGT group” (species with higher OGT within the genus) and a “low OGT group” (species with lower OGT within the genus). We chose high and low OGT groups by iteratively extracting pairs of species with the highest and lowest OGT in a focal genus until the difference was less than 10 degrees, with a maximum of three pairs per genus to alleviate phylogenetic bias. We then performed Fisher’s exact test to search for candidate genes associated with OGT (see Materials and Methods). Although genes broadly contributing to the change of OGT across various taxa were rare, K13993 (heat shock protein 20), which is known to protect proteins against heat-induced denaturation and aggregation (33), was significantly more often present in the high OGT group compared with the low OGT group (*P* = 4e−6 [0.04 after Bonferroni correction]) (Fig. 4a; Table S2). The phylogenetic analysis revealed that K13393 was widely distributed among bacterial lineages, suggesting its recurrent gains and losses (Fig. 4b). To examine how the gains and losses of K13993 affect OGT, we inferred the ancestral state of the gene presence/absence. We compared it with the OGT of the ancestral species, finding that the loss of K13993 was associated with the decrease of OGT (−3.6°C on average), although the acquisition of K13993 had little influence on OGT (−0.1°C on average).

Although genes specific to cold adaptation are likely critical for improving the prediction of OGT in psychrophiles, such genes might not have been detected by the above method because of the limited number of psychrophiles in the data set. To overcome this, we also searched for genes present in psychrophiles by comparing them with other bacteria (Table 1; Table S3). The presence/absence of the K06970 [23S rRNA (adenine1618-N6)-methyltransferase] gene showed the strongest association (*P* = 5e−34 [5e−30 after Bonferroni correction]). This gene is known to help maintain the ribosomal peptide tunnel in the proper conformation (34), suggesting that it can play an important role in structural stability at low temperatures. We confirmed the acquisition and the loss of K06970 was associated with the decrease (−2.5°C on average) and the increase of OGT (2.7°C on average), respectively. Furthermore, we found that the presence of two electron transport chain-related genes (K18159 and K08325) was also associated with cold temperature (Table 1). Those genes are suggested to associate the metabolic changes in low temperature environments (1, 35). The pathway enrichment analysis of the top 50 genes revealed that psychrophiles tend to possess specific pathways related to anaerobic metabolism, such as methane and propionate metabolism (Fig. S4). In contrast, psychrophiles tended to lose genes in the aerobic processes, such as pyruvate metabolism and the tricarboxylic acid cycle, possibly associated with the cold and anaerobic deep sea environments (36). These results suggest that gene presence/absence information is a promising feature for capturing OGT changes that are not fully reflected in the genomic sequence optimization.

**TABLE 1:** Top 10 candidate genes associated with changes in OGT across diverse lineages^*a*^

KEGG ortholog	*P*-value	*P*-value_less (specifically present in “low OGT group”)	*P*-value_greater (specifically present in “high OGT group”)	Description
K13993	4.35.E−06	1.00.E+00	2.18.E−0	HSP20; HSP20 family protein
K07498	2.14.E−05	1.07.E-05	1.00.E+00	K07498; putative transposase
K22024	1.49.E−03	1.00.E+00	7.45.E−04	pdxA2; 4-phospho-D-threonate 3-dehydrogenase/4-phospho-D-erythronate 3-dehydrogenase (EC 1.1.1.408, EC 1.1.1.409)
K22129	1.73.E−03	1.00.E+00	8.65.E−04	dtnK, denK; D-threonate/D-erythronate kinase (EC 2.7.1.219, EC 2.7.1.220)
K25495	1.87.E−03	9.35.E−04	1.00.E+00	nimR; AraC family transcriptional regulator, regulator of nimT
K04095	2.39.E−03	1.20.E−03	9.99.E−01	fic, FICD, HYPE; cell filamentation protein, protein adenylyltransferase (EC 2.7.7.108)
K22684	3.45.E−03	1.72.E−03	1.00.E+00	MCA1; metacaspase-1 (EC 3.4.22.-)
K07482	4.81.E−03	2.41.E−03	9.99.E−01	K07482; transposase, IS30 family
K07178	4.98.E−03	2.49.E−03	9.99.E−01	RIOK1; RIO kinase 1 (EC 2.7.11.1)
K00298	6.29.E−03	3.14.E−03	1.00.E+00	ceo; N5-(carboxyethyl)ornithine synthase (EC 1.5.1.24)

^*a*^ KO numbers, *P*-values (with Bonferroni correction for multiple comparisons, significance level = 5.0E−6 [0.05/9959]), and descriptions for the top 10 genes are shown. Genes that are significantly present in either group were tested using Fisher’s exact test.

To evaluate whether incorporating gene presence/absence information improves the OGT prediction, we included the gene copy number information of the top 50 genes identified by each of the two methods described above (100 genes in total). For each training data set, gene lists were generated independently to ensure no information was used from the phylum of the test data. By incorporating the gene copy number information, the accuracy in the prediction for the OGT of psychrophiles was improved (mean absolute error of psychrophiles = 6.3°C) (Fig. 4c through e), particularly in the species with OGT < 5°C (mean improvement = 7.6°C). In the Lasso model, 83 out of the 100 genes were retained with non-zero coefficients, and 16 of the top 100 features with the largest absolute coefficients were gene copy features (Table S4). When compared with the prediction solely based on genome composition, the model demonstrated enhanced performance (1.1°C on average) for species experiencing a rapid OGT shift (dOGT ≤ −10°C and 10°C ≤ dOGT) (Fig. 4e). Conversely, accuracy did not improve for species whose absolute dOGT was less than 10°C (−0.86°C on average; Fig. 4e), resulting in a slight decrease in the overall accuracy (*R*^2^ = 0.80). These results confirm that gene acquisition and loss significantly influenced the changes in OGT and demonstrate that incorporating gene presence/absence information improves the prediction of species experiencing the rapid OGT shift, especially psychrophiles. Finally, we investigated how accurately our model can classify psychrophilic species. We found that, while the previous model correctly classified only 63 out of 164 psychrophiles (38.4%) as psychrophilic, the classification performance of the new model was increased to 80 out of 164 (48.7%) (Fig. S5). We note that the number of non-psychrophilic species misclassified as psychrophiles increased from 2.2% (33 out of 1467) to 6.1% (90 out of 1467).

### The conservation of OGT influences the correlation between OGT and genomic features

**Fig 5:**
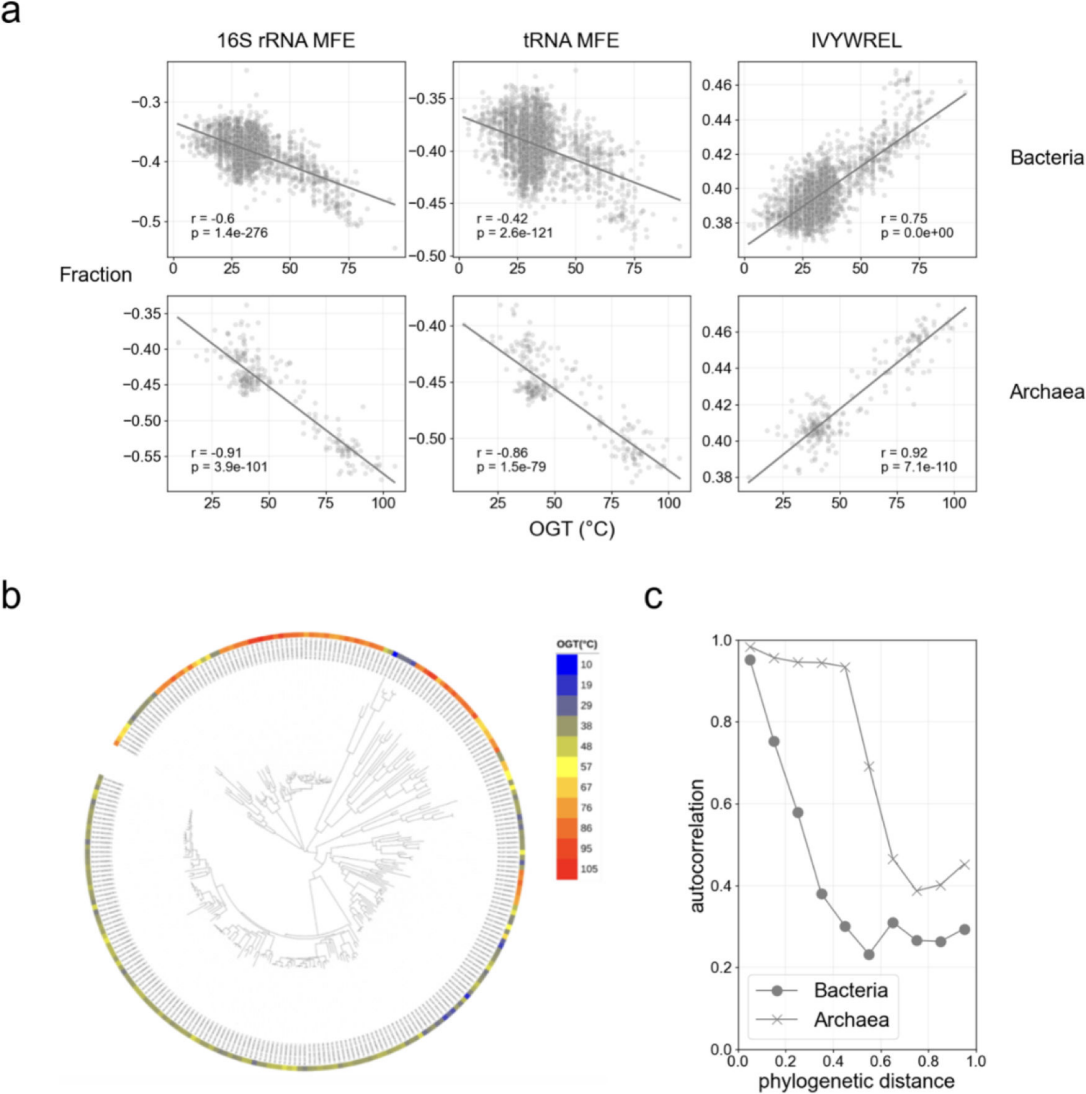
The conservation of OGT in bacteria and archaea. (**a**) The relationship between the genomic features (tRNA MFE, rRNA MFE, and summation of IVYWREL) and OGT. The top and bottom rows show bacteria and archaea, respectively. Pearson’s correlation coefficients and the *P*-values of the no-correlation test are shown. (**b**) The distribution of OGT of archaea in the phylogenetic tree obtained from GTDB r207. The color represents OGT. (**c**) The phylogenetic conservation of OGT of bacteria and archaea represented by autocorrelation in the tree. The circle and cross marks represent bacteria and archaea, respectively.

A previous study reported higher feature correlations with OGT in archaea than in bacteria, which makes archaea’s OGT more predictable (12), partly because of the wide range of OGT in bacteria. However, the relationship of feature correlation with the conservation of OGT of bacteria and archaea has not been sufficiently explored. Because rapid alterations in OGT influence the accuracy of prediction, as we demonstrated, the differences in the phylogenetic conservation of OGTs between archaea and bacteria would also be a key to determining the accuracy. Here, we compared OGT and genomic features (MFE of 16s rRNA, MFE of tRNA, and amino acid composition IVYWREL) in bacteria and archaea (Fig. 5a). To investigate the conservation of OGT in archaea, we mapped the OGT in archaea to the phylogenetic tree and compared the trait autocorrelation of bacteria and archaea (Fig. 5b and c), finding that OGT was more conserved and stable in archaea than in bacteria. As previously reported (12), the correlation between OGT and genomic features was stronger in archaea than in bacteria (see Table S5 for the correlations between all features and OGT of bacteria and archaea). Although the majority of features (434/526) correlated more strongly with OGT in archaea than in bacteria, some features, such as the glutamic acid ratio, showed a higher correlation coefficient in bacteria than in archaea, suggesting differences in their contributions to temperature adaptation (Table S5).

## DISCUSSION

In this study, we demonstrated that incorporating gene presence/absence information into our model enhances the prediction of psychrophiles, which are often difficult to predict using genome composition alone (12) (e.g., base composition, codon composition, and amino acid composition). Although genomic data from uncultivated prokaryotes have exponentially increased (11), we do not know how many psychrophiles are hidden in those data. The ability to predict previously unknown psychrophiles may contribute to the discovery of new cold adaptation mechanisms and the identification of enzymes that remain active at low temperatures. Previous studies typically separated training and test data randomly, validating accuracy in this manner (14, 18). However, since uncultured bacteria often belong to clades that are distinct from cultured bacteria (37), it would be more appropriate to validate accuracy for clades not present in the training data. In this study, we employed leave-one-phylum-out cross-validation to ensure our model could predict the OGT of phyla absent from the training data. Interestingly, the linear model outperformed the non-linear model (SVR) under these conditions, indicating that the robustness of the linear model allowed it to capture genome composition features that are thermodynamically more meaningful. Those results suggest that our model may be applicable to rarely cultivated lineages such as candidate phyla radiation (38).

The results suggest that the challenges in predicting psychrophiles primarily arise from the rapid decline in OGT from their ancestors. OGT can shift rapidly because of horizontal gene transfer or single nucleotide mutations (1, 39–42), as laboratory evolution experiments have demonstrated (40, 41). Contrary to this, changes in the genome composition often require the accumulation of numerous mutations and take considerable time (29, 30). We propose this difference in the mechanism of temperature adaptation causes the delay in the adaptation of genome composition to OGT, which influences the accuracy of predictions based on genome composition. Our data set is not ideal to test this idea because it does not include “old” psychrophiles that have been psychrophiles since the common ancestors. But, we note a recent study consistent with this idea, suggesting that optimization of amino acid composition can also occur in psychrophiles over a long period when its lineage is ancestrally cold-adapted (30). From this perspective, even though we identified features around the start codon reflecting cold adaptation (Fig. 1c), these may not have undergone sufficient optimization. This could explain why incorporating features around start codons did not significantly enhance the prediction accuracy of psychrophiles. The results suggest that information on gene acquisition and loss is valuable for predicting OGT diversity within genera, leading to the improvement of the prediction of OGT of psychrophiles. While some of the genes whose presence/absence was associated with OGT could be directly involved in temperature adaptation, many of them are functionally uncharacterized, which requires further experimental investigation. We note that not only presence/absence but also single nucleotide substitutions can contribute to the changes in OGT (40, 41), and future models could incorporate their effects into the predictions, potentially improving accuracy. While we focused on OGT in this study, similar challenges may arise in predicting rapidly altered phenotypes for other traits, such as optimal pH and salinity, as these are also known to be influenced by the gain and loss of specific genes (29, 43). The present study suggests that gene information may also be important for predicting such phenotypes.

Previous studies discussed that the OGT of archaea is more accurately predictable than those of bacteria (12). Although this phenomenon can be caused by multiple factors, such as the range of OGT (12, 44) and a relatively low fraction of psychrophiles in archaea, our finding of the high conservation of OGT in archaea suggests that their genomes have undergone long-term optimization. A previous study suggested that evolution in archaea tends to be relatively stable because of their inherent advantages in adapting to chronic energy stress, whereas bacterial evolution is more dynamic (45).

We note a few caveats of this study. First, with our leave-one-phylum-out cross-validation, it is not practical to perform CV solely on the training data and to evaluate the accuracy using a separated test data. Since we report the results of CV conducted on the entire data set in this study, the parameter selection process in the evaluation may cause a slight overestimation. Second, the non-uniformity of data quality across OGT may influence the model performance. In general, extremophiles exhibit slow growth rates (46), thus their observed OGT may be less accurate.

In our study, we proposed that a model based on genome composition poses a challenge in predicting the OGT of species whose OGT has rapidly changed, and that combining gene presence/absence information would be the key to predicting the OGT of psychrophiles. Although machine-learning models based on genome composition for phenotype prediction have gained attention in recent years (12, 14, 47), the present study highlights the importance of incorporating gene sets related to phenotype into predictions.

## MATERIALS AND METHODS

### Data set of OGT and genome

Bacterial and archaeal OGT data were obtained from three publicly available data sets (I: 11,004 species (18), II: 8,639 species (16), and III: 4,663 species (17). All data were downloaded on 3 October 2022. Most of these OGT data are experimentally determined optimal incubation temperatures. The species with OGTs exactly matching 25°C, 28°C, 30°C, and 37°C were most frequently observed in all three databases. We excluded those species from the analysis because these temperatures are often used as conventional incubation temperatures in laboratories and seem to be an artifact caused by hand-built bias (48). As a result, the number of species was 4,038 for the data set I, 4,312 for II, and 2,527 for III. The three databases were merged, and overlapping species were averaged. The merged data resulted in 8,972 species. The OGT data were merged with representative genomes from the Genome Taxonomy Database (GTDB) r207 (19–22) with completeness >95% and contamination <5% using the species name as a key because OGT data with genome accession were only available for 7% of species (637 species). Using OGT data of the species with more than three strains (17), we ensured that the diversity of OGT within species was relatively small (median of standard deviation = 0.52°C).

Coding sequence annotations were downloaded from GTDB r207 with data predicted by Prodigal 2.6.3 (49). tRNA annotation was performed using tRNAscan-SE 2.0.9 (50). and rRNA annotation was performed using barrnap 0.9 (https://github.com/tseemann/barrnap). Excluding species for which some of the 5S rRNA, 16S rRNA, and 23S rRNA annotations were missing, the data of 2,869 bacterial and 262 archaeal species were used in this study. The taxonomy of each species was defined based on GTDB taxonomy. The phylogenetic tree was obtained from GTDB r207 and was drawn using iTOL (interactive Tree of Life) v6 (51). For rRNA and tRNA, the most stable structures and minimum free energies were obtained using Linearfold (52) (parameters were set to -V and a Vienna RNAfold-based model was used).

### Downsampling of the mesophiles

The distribution of bacterial OGT is highly biased toward the mid-temperature range (20°C to 40°C). Therefore, we also made the downsampled data set of bacteria (Fig. 1a). For the downsampling, the species with the highest genome quality score (completeness − 5 × contamination) were sampled from each genus for species with OGT between 20°C and 40°C. With this downsampling, 1,631 species were kept, and training and evaluation of regressions were conducted using this data set. To avoid the influence on the biological interpretation, the prediction result of all data were used for the following analysis.

### Extraction of specific information near start codons

The following method was used to extract site-specific changes near the start codon. *F_i_* is the features of the average base composition (*i*-th to *i*+9th) and amino acid composition (*i*-th) counting from the start codon. The mean value of all genes was calculated for each species. To extract the site-specific information of *F*_*i*_, we normalized *F*_*i*_ using the representative value of *F*_rep_:


(1) $$F_{i,normalized}=\log_{2}\left(F_{i}/F_{rep}\right)$$

The *F*_rep_ value was the 50th value from the start codon when analyzing base composition, and the average of total length sequence when analyzing amino acid composition. We used *F*_5_ as the representative feature for guanine composition near the start codon, and the mean of *F*_1_–*F*_4_ as the representative feature for amino acid composition near the start codon.

### Creating regression models based on genome composition

 We calculated a total of 526 features from the genomes (Table 1). All features were normalized to a maximum value of 1 and a minimum value of 0. For machine learning, Lasso regression and SVR were used (Scikit-learn 1.2.2). To verify the accuracy for unknown clades, leave-one-phylum-out cross-validation was used, in which one phylum was the test data, and the rest were the training data, and validation was performed so that all phyla were the test data one by one. Root mean squared error (RMSE) and coefficient of determination (*R*²) were used to evaluate the prediction accuracy. The parameter yielding the highest *R*² score in the leave-one-phylum-out cross-validation was selected for the final model (Lasso: alpha = {0.001, 0.002, 0.004, 0.006, 0.008, 0.01}, SVR: C = {0.1, 1, 10, 100}, epsilon = {0.001, 0.01, 0.1}, others = default values). These parameters were kept the same in the model after incorporating presence/absence gene information to facilitate a straightforward comparison.

### Ancestral state reconstruction of OGT and genes

The OGT of the most recent common ancestor of each genus was estimated by minimizing the summation of squared changes in OGT along the branches of the tree (“asr_squared_change_parsimony()” function from R package castor 1.7.11 with the parameter “weighted” set to True). By calculating the difference between the OGT of the common ancestor of each clade (with more than five species) and the OGT of the current species (OGT_current_species_ − OGT_common_ancestor_), we calculated how much the OGT of that species has changed from the ancestor (dOGT). Similarly, the ancestral state of genes (presence or absence) was inferred using the function “asr_mk_model ()” function. The trait autocorrelation of OGT was calculated using the “get_trait_acf()” function.

### Annotation of gene and gene set enrichment analysis

The CDSs of the genome were functionally annotated using KofamScan version 1.3.0 with default parameters (53). For the best functional prediction, the default asterisk annotation was adopted. To search for genes related to OGT, we focused on genera that included both high and low OGT species. Specifically, pairs of species with the highest and lowest OGT in a focal genus were extracted iteratively until the difference was less than 10 degrees, with a maximum of three pairs per genus to alleviate phylogenetic bias. We then performed Fisher’s exact tests with Bonferroni corrections to assess the statistical significance of the association between OGT and the gene presence/absence. Similarly, we performed Fisher’s exact tests for psychrophiles (OGT < 20°C) and other bacteria under the same criteria. KEGG pathway enrichment analysis was conducted using R package clusterProfiler version 4.14.4 (54). All codes used in this study are available at https://github.com/tsuchimatsu/OGT_prediction.

## Data Availability

All codes used in this study are available at https://github.com/tsuchimatsu/OGT_prediction.

## SUPPLEMENTAL MATERIAL

Supplemental figures msystems.00062-26-s0001.docx Fig. S1 to S5.

Supplemental tables msystems.00062-26-s0002.xlsx Tables S1 to S5.

ASM does not own the copyrights to Supplemental Material that may be linked to, or accessed through, an article. The authors have granted ASM a non-exclusive, world-wide license to publish the Supplemental Material files. Please contact the corresponding author directly for reuse.
