# Supplementary material for: Genomic and evolutionary factors influencing the prediction accuracy of optimal growth temperature in prokaryotes
Source: mSystems. 2026 Apr 3;11(5):e00062-26. doi: 10.1128/msystems.00062-26 (PMC13185595; doi:10.1128/msystems.00062-26)
Supplement: Supplemental figures — Fig. S1 to S5. [file msystems.00062-26-s0001.docx]

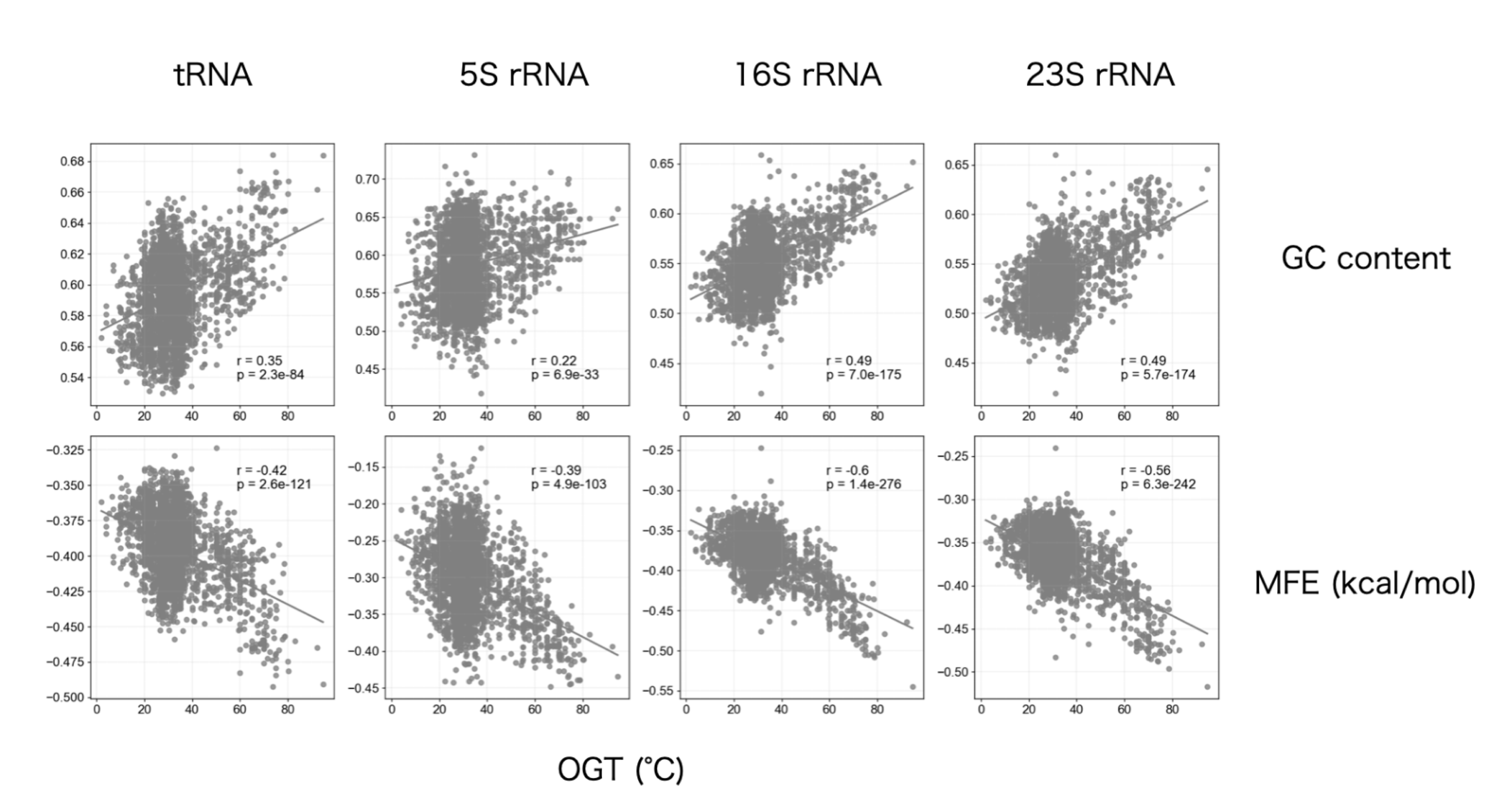


**Supplementary Figure 1. The relationship between OGT, GC content, and MFE of tRNA and rRNA.** MFE of tRNA and rRNA were normalized by sequence length. Pearson’s correlation coefficients and the p-values of the no-correlation test are shown.


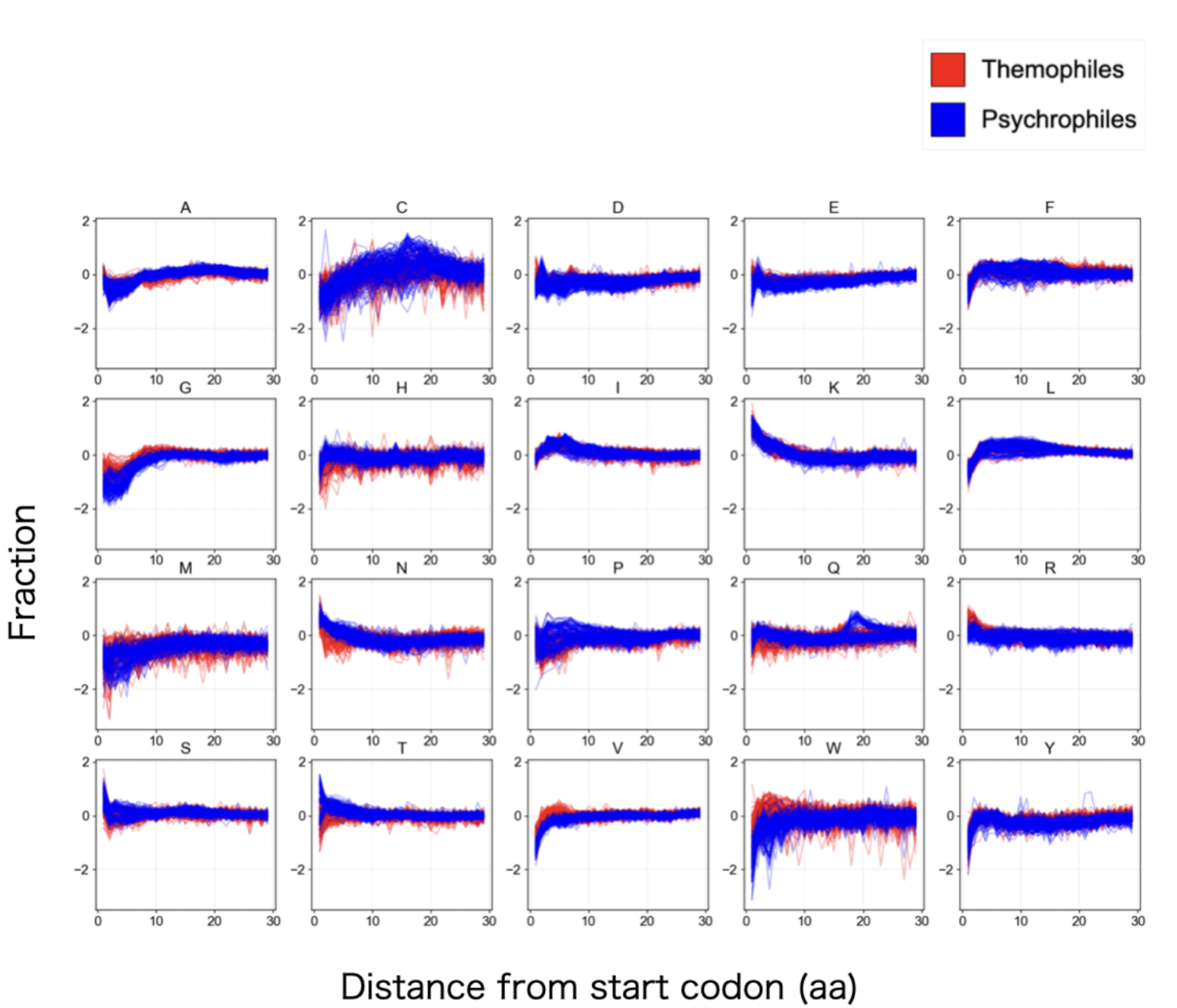


**Supplementary Figure 2. The fraction of amino acids in the downstream region of the start codon.** The fraction was normalized by the average of the fraction of each amino acid of species. The red and blue lines indicate thermophile and psychrophile, respectively.


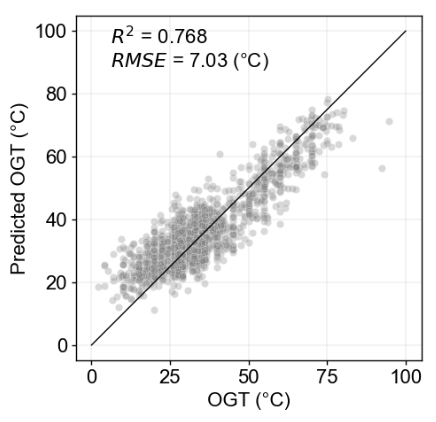


**Supplementary Figure 3. The predictions based on genome composition using SVR.** Each dot represents one bacterial species.


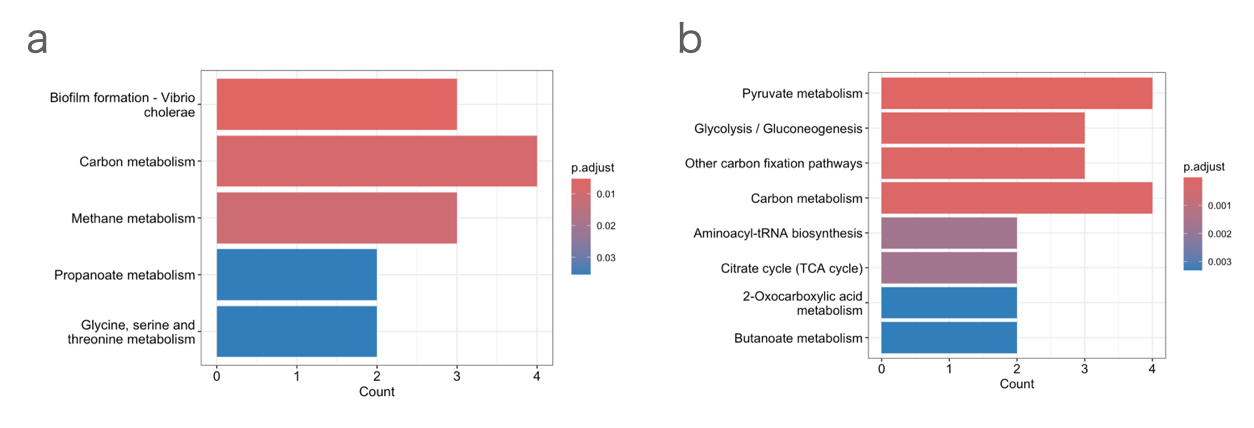


**Supplementary Figure 4. The pathway enrichment analysis of the top 50 cold adaptation-associated genes (adjusted p-value < 0.01).** Enrichment pathways with genes that are specifically (a) present or (b) absent in psychrophilces.


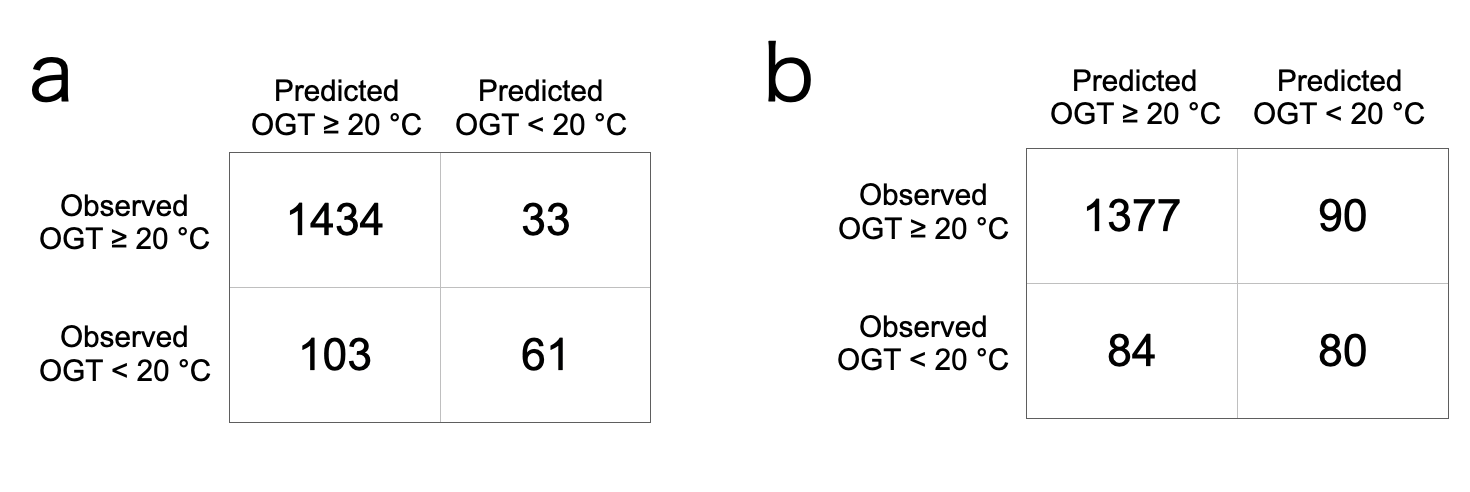


**Supplementary Figure 5. Improvement in the classification of psychrophiles by incorporating the presence/absence of OGT-associated genes.** Each panel shows the confusion matrix of psychrophile classification: (a) before and (b) after including OGT-associated gene information.
